# SARS-CoV-2 seroprevalence around the world: an updated systematic review and meta-analysis

**DOI:** 10.1186/s40001-022-00710-2

**Published:** 2022-06-02

**Authors:** Mobin Azami, Yousef Moradi, Asra Moradkhani, Abbas Aghaei

**Affiliations:** 1 Student Research Committee, Kurdistan University of Medical Sciences, Sanandaj, Iran; 2 Department of Epidemiology and Biostatistics, Faculty of Medicine, Kurdistan University of Medical Science, Sanandaj, Iran; 3 Social Determinants of Health Research Center, Research Institute for Health Development, Kurdistan University of Medical Sciences, Sanandaj, Iran

**Keywords:** Covid-19, SARS-CoV-2, Global seroprevalence, Serum antibodies (IgG and/or IgM), Systematic review, Meta-analysis

## Abstract

**Background:**

Covid-19 has been one of the major concerns around the world in the last 2 years. One of the challenges of this disease has been to determine its prevalence. Conflicting results of the serology test in Covid explored the need for an updated meta-analysis on this issue. Thus, this systematic review aimed to estimate the prevalence of global SARS-CoV-2 serology in different populations and geographical areas.

**Methods:**

To identify studies evaluating the seroprevalence of SARS-CoV-2, a comprehensive literature search was performed from international databases, including Medline (PubMed), Web of Sciences, Scopus, EMBASE, and CINHAL.

**Results:**

In this meta-analysis, the results showed that SARS-CoV-2 seroprevalence is between 3 and 15% worldwide. In Eastern Mediterranean, the pooled estimate of seroprevalence SARS-CoV-2 was 15% (CI 95% 5–29%), and in Africa, the pooled estimate was 6% (CI 95% 1–13%). In America, the pooled estimate was 8% (CI 95% 6–11%), and in Europe, the pooled estimate was 5% (CI 95% 4–6%). Also the last region, Western Pacific, the pooled estimate was 3% (CI 95% 2–4%). Besides, we analyzed three of these areas separately. This analysis estimated the prevalence in subgroups such as study population, diagnostic methods, sampling methods, time, perspective, and type of the study.

**Conclusion:**

The present meta-analysis showed that the seroprevalence of SARS-CoV-2 has been between 3 and 15% worldwide. Even considering the low estimate of this rate and the increasing vaccination in the world, many people are still susceptible to SARS-CoV-2.

## Background

Scientists first reported infection due to severe acute respiratory syndrome coronavirus 2 (SARS-CoV-2) in Wuhan, China, in December 2019 [1], and due to its contagious nature, it rapidly spread throughout China and the world as the WHO declared a pandemic on March 11, 2020 [2, 3]. According to the World Health Organization (WHO), more than 220 million cases have been identified worldwide; more than 5 million have died [4]. The presented statistics show only a part of the total cases because the clinical manifestations of patients with SARS-CoV-2 vary from acute diseases with severe pneumonia, acute respiratory distress syndrome, or multiple organ failure up to asymptomatic infection. Asymptomatic carriers are essential sources of the infection spread during the incubation period and interfere with the prevention and control of the disease. So, this group of people is an important challenge in the current management of the pandemic [5–7].

The ideal method for detecting Covid-19 is a real-time reverse transcription-polymerase chain reaction (RT-PCR). Still, the disease may not be detectable for various reasons, including low viral concentrations in the upper respiratory tract, non-standard sampling methods, and reduced viral load one week after the onset of symptoms. False-negative results may be reported [3, 8]. However, because SARS-COV-2 infection can induce innate and acquired immunity, resulting in widespread inflammatory responses in the disease [9], and neutralizing antibodies (Nabs) made against spike glycoprotein or SARS-CoV-2 nucleocapsid protein are often lead to a long-term immune response in viral infections which in most patients with different titers can be detected within 14 to 21 days after the onset of symptoms and at least for several months thereafter [8, 10], the method of serological testing replaces and complements molecular testing by detecting virus-specific antibodies in blood samples such as IgM and IgG and through commercially available tests including lateral flow immunoassays (LFIAs), enzyme-linked immunoassays (ELISAs), fluorescence immunoassays (FIA), chemiluminescence assays (CLIAs), electro-chemiluminescent immunoassay (ECLIA), and pseudovirus neutralization assays (PsVN assay or VN), and it is used to estimate the serum prevalence in the population and thus the total number of previous infections to diagnose asymptomatic cases, post-clinical convalescence, post-vaccine responses and as a diagnostic aid method in false-negative cases reported by PCR [11–13].

To date, epidemiologists from many countries conducted seroprevalence studies on different populations. The results are significantly different between studies, and in many cases, the actual number of patients is higher than the recorded cases. Therefore, they cannot be the exact measure of serum prevalence in the general population and the true extent of pandemic dynamics. As a result, differences in the presented statistics can lead to inappropriate policies and harm to public health [7, 8, 10]. Because Covid-19 has become a global threat and its spread depends on social interactions, population density, education, health promotion, and other related factors, determining the prevalence of infection and collective immunity against SARS-CoV-2 and the use of these data are necessary for making decisions about control measures, management, and assessment of epidemic risks. Therefore, in this meta-analysis, we aimed to estimate the prevalence of global SARS-CoV-2 serology in different populations and geographical areas and investigate the factors affecting it.

## Methods

This systematic review and meta-analysis were based on PRISMA guidelines which are specific to the systematic review and meta-analysis of observational studies [14, 15].

### Search strategy

All original articles published from December 2019 to December 2021 were searched without language restrictions in international databases, including Medline (PubMed), Web of Sciences, Scopus, EMBASE, and CINHAL. The search strategy in this study was performed using the main study keywords, including serologic tests (with synonyms of serologic, serology, serology studies) SARS-CoV-2 (with synonyms of Covid-19).

Gray Literature was then searched to access unpublished articles and dissertations or international reports. In addition, after the final selection of articles, a manual search was performed by reviewing the references of related articles. Also, medrxiv and bioRxiv websites were used for findings preprint studies related to seroprevalence of SARS-CoV-2 from inception to December 2021.

### Study selection and eligibility criteria

The search strategy in international databases was independently performed by the two researchers (MA and AM), and the disputes were resolved by the third person (YM).

#### Inclusion criteria

In this meta-analysis, studies were considered whose main purpose was to determine the prevalence of positive serological tests in different communities; that is, after performing tests at different times in other communities, the prevalence of the number of positive tests was examined. Therefore, cohort and cross-sectional studies were included in this meta-analysis. The statistical population studied in these initial articles were all individuals, whether with a specific disease or healthy. There were no particular restrictions on the method of serological diagnosis of Covid-19 in this study for inclusion of studies, and various serological tests such as ELISA, LFIA, VN, CLIA, and ECLIA were included in the research. The definition of Covid-19 disease in this study was based on its international definition affected by the transmission of the SARS-CoV-2 virus.

#### Exclusion criteria

Other studies, including case reports or case series, systematic reviews, and meta-analyses, as well as letters or editorials, were excluded from this study.

### Data extraction

To extract information, first, a checklist including questions on the first author’s name, date of publication, country, WHO region, type of sampling (random or non-random), duration of the study, type of the serological test, race, and ethnicity, age, gender (male, and female), number of positive tests and number of performed tests was designed. Then, information extraction based on the checklist was independently performed by the two authors (AM and MA), and disputes, if any, were resolved by the third person (YM).

### Quality assessment

In this study, to evaluate the quality of included articles, the Joanna Briggs Institute (JBI) critical appraisal checklist was used for observational studies. JBI critical appraisal tools have been developed by the JBI and collaborators and approved by the JBI Scientific Committee following extensive peer review.

### Statistical analysis

According to the extracted information, the Metaprop command was used to calculate the pooled prevalence, and the results were analysed [16]. Cochrane Q and I2 tests were used to investigate the heterogeneity and variance between the studies selected for meta-analysis [17–20]. Funnel Plot and Egger test were used to evaluate the publication bias [19, 20]. Also, the meta-regression analysis and diagram were used to examine the association between important variables with the estimated pooled prevalence. Statistical analysis was performed using STATA 16.0.

## Results

**Fig. 1:**
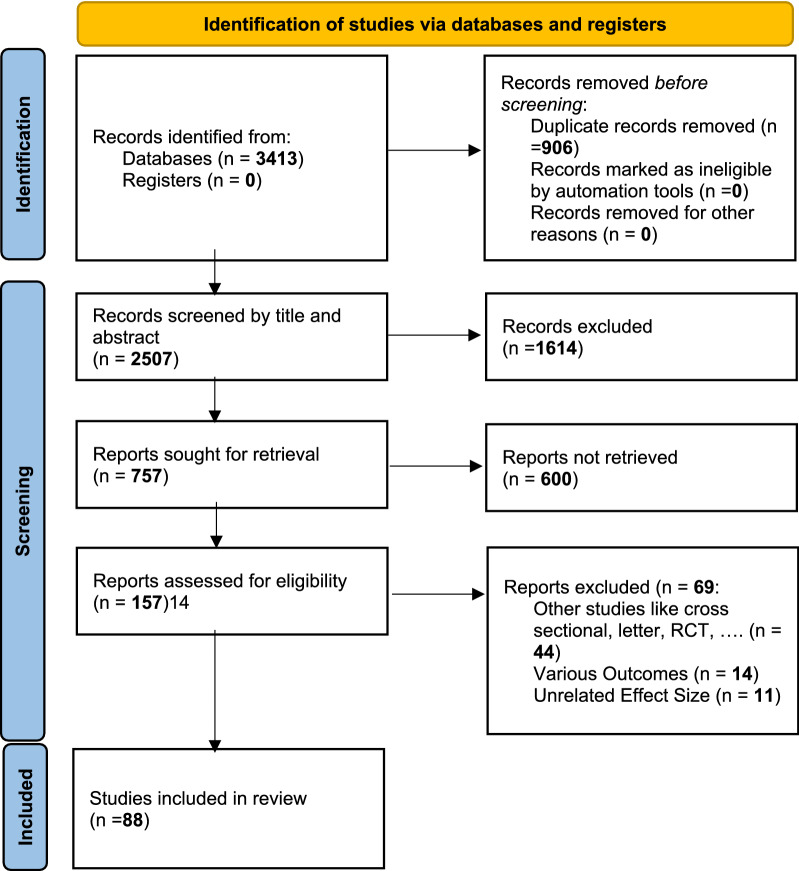
PRISMA 2020 flow diagram for new systematic reviews, which included searches of databases and registers only

As a result of searching the electronic databases, 3413 studies were obtained, and after removing duplicates, 2507 studies remained. After eliminating studies conducted before 2019, 1926 titles remained for review. In the last stage, after reviewing titles, abstracts, and full texts and considering the inclusion and exclusion criteria, 88 studies were selected for inclusion in the study (Fig. 1).

All 88 studies entered at different time intervals examined the prevalence of positive tests in various communities (Table 1). In total, 414,773 serological tests were performed in all studies. Studies have been reviewed in different countries and were also divided according to WHO classifications. In total, studies have been conducted in 34 countries, with 26 in the United States, 7 in Italy, 5 in France, 4 in each country of Japan, the United Kingdom, Brazil, and China, 3 in each country of Spain, Germany, and Denmark, and 2 in each country of Belgium, Iran, Greece, and Sweden, and 1 in each one of the other countries. According to the WHO classification, there were four studies in the Eastern Mediterranean, 4 in Africa, 31 in America, 35 in Europe, and 12 in Western Pacific.

**Table 1:** Characteristics of included studies

Authors (years) (R)	Country/WHO Regions	Study population	Sampling methods (random or non-random)	Study period	Type of detection methods	Race/ethnicity		Gender Male		Age		Seropositive people (based on months)	No. of people screened (sample size)	Seropositive people (total)
Herzog et al. [40]	Belgium (European Region)	Individuals aged 0–101 years	Random	March–July, 2020	ELISA			1799 (46.0%)		Highest % 60-70Y 507 (13.0%)		30 March–5 April 113	16,532	840
								1599 (47.1%)		10-20Y 442 (13.0%)		20-26April 204		
								1587 (49.0%)		10-20Y 431 (13.3%)		18–26 May 224		
								1425 (48.1%)		60-70Y 399 (13.5%)		8–13 June 163		
								1471 (48.7%)		10-20Y 413 (13.7%)		29 June–4 July 136		
Filho et al. [41]	Brazil (Region of the Americas)	Blood donors in Rio de Janeiro	Non-random	April, 2020	LFIA			1450		Highest % 30–49Y 1443 (3.7%)			2857	114 (4.0%)
Silveira et al. [42]	Brazil (region of the Americas)	Individuals in Canoas, Caxias do Sul, Ijuí, Passo Fundo, Pelotas, Porto Alegre, Santa Cruz do Sul, Santa Maria and Uruguaiana	Random (multi-stage sampling)	March–May, 2020	LFIA	White 76.0%		41.1%		Highest % 50–59 (17.1%)			4500	18
						Brown 15.3%								
						Black 7.4%								
						Other 1.3%								
Torres et al. [43]	Chile (Region of the Americas)	Large School Community Subject	Non-random	April, 2020	LFIA			Students 54%		Mean 10.8			1009	100
								Staff 27%		42.8			235	39
Chang et al. [44]	China (Western Pacific Region)	Blood donors in the cities of Wuhan, Shenzhen, and Shijiazhuang among 18–60-year-old adults	Non-random	January–April, 2020	VN	Wuhan Han: 17,126 (96.2)		11,077 (62.3)		Median 33			17,794	515
						Non-Han: 533 (3.0)								
						Missing data: 135 (0.8)								
						Shenzhen Han: 6519 (95.7)		4428 (65.0)		36			6810	3
						Non-Han: 274 (4.0)								
						Missing data: 17 (0.2)								
						Shijiazhuang Han: 13,414 (99.1)		9542 (70.5)		40			13,540	1
						Non-Han: 124 (0.9)								
						Missing data: 2 (0.0)								
To et al. [45]	China (Western Pacific Region)	In a hospital and university in Hong Kong	Random	December, 2019-Februray, 2020	ELISA					Median 59		April 12 to July 3, 2018: 295 (P7)	1214	29
												Jan 2 to June 28, 2019: 429 (17)		
												July 2 to Dec 31, 2019: 401 (13)		
												Jan 1 to Jan 31, 2020: 580 (15)		
												Feb 1 to Feb 13, 2020: 233 (1)		
Liang et al. [46]	China (Western Pacific Region)	Hospital visitors	Random	January–April, 2020	CLIA			Wuhan 4140 (50.0)		Median 55			8272	174
								Guangzhou 4249 (48.3)		54			8782	53
Jerković et al. [47]	Croatia (European Region)	In Industry workers in Split-Dalmatia and Sˇibenik-Knin	Non-random	April, 2020	LFIA			Split-Dalmatia		Median 46			1316	13
								Knin		45			178	6
Erikstrup et al. [48]	Denmark (European Region)	Blood donors aged 17–69 years	Non-random	April–May, 2020	LFIA			10,217		Highest % 5068			20,640	412
Petersen et al. [49]	Denmark (European Region)	Individuals In Faroe Islands	Random	April–May, 2020	ELISA			538 (50.2)		Median 42.1			1075	6
Ward et al. [50]	England (European Region)	ages 18 + years in England	Non-random	June–July, 2020	LFIA	White: 92,737		43,825		Highest % 45–54 20,634			99,908	5544
						Mixed: 1347								
						Asian: 3658								
						Black: 900								
						Other: 762								
Gallian et al. [51]	France (European Region)	In group O French blood donors	Non-random	March–April, 2020	VN			534		Median 41			998	27
Grzelak et al. [52]	France (European Region)	hospitalized patients, pauci-symptomatic individuals and blood donors	Random	March, 2020	ELISA			70 (35%)		Median 18			200	3
Fischer et al. [53]	Germany (European Region)	In blood donors located in three different federal states	Non-random	March–-June, 2020	ELISA								3186	29
Weis et al. [54]	Germany (European Region)	Individuals The CoNAN study	Non-random	May, 2020	ELISA			266 (47.3%)		Median 60			562	51
Bogogian-nidou et a. [55]	Greece (European Region)	Greece People by using the leftover sampling methodology	Random	March–April, 2020	CLIA			3001				March 5	6586	24
												April 19		
Merkely et al. [56]	Hungary (European Region)	Hungarian population included individuals aged 14 years or older, living in private households	Random	May, 2020	CLIA			4864 (46.4)		Mean 48.7			10,474	69
Shakiba et al. [57]	Iran (Eastern Mediterranean Region)	Individuals in Guilan province, Iran	Random	April, 2020	LFIA			270(49)		Highest % 18–60 343			551	117
Percivalle et al. [58]	Italy (European Region)	In blood donors from the Lodi Red Zone in Lombardy, Italy	Non-random	January–February, 2020	VN			272 (70%)		Median 43			390	91
Valenti et al. [59]	Italy (European Region)	Blood donors during the Covid-19 Milan outbreak	Random	February–April, 2020	LFIA			453		Mean 40.7			729	40
Fiore et al. [60]	Italy (European Region)	In healthy blood donors in South Eastern Italy	Random	May, 2020	CLIA			665		Highest % (46‐55) 246			904	9
Doi et al. [61]	Japan (Western Pacific Region)	Individuals in Kobe, Japan	Random	March–April, 2020	LFIA			486		Highest % 60–69 171			1000	33
Takita et al. [62]	Japan (Western Pacific Region)	Individuals in primary care clinics in Tokyo, Japan	Random	March–April, 2020	LFIA			461		Highest % 35–54 653			1071	41
Takita et al. [63]	Japan (Western Pacific Region)	Individuals at community clinics in Tokyo Authors:	Non-random	April–May, 2020	LFIA			87 (59%)		Highest % 40–49 58 (39)			147	7
Uyoga et al. [64]	Kenya (African Region)	In Kenyan blood donors	Random	April–June, 2020	ELISA			2540		Highest % 25 to 34 1242			3098	174
Song et al. [65]	Korea (Western Pacific Region)	Individuals without a history of the coronavirus disease infection in Daegu, Korea	Random	May–June, 2020	LFIA			99 (50%)		Highest % 40–59 89			198	15(7.6)
Kammon et al. [66]	Libya (African Region)	Among public community and health-care workers in Alzintan City of Libya	Random	April–May, 2020	LFIA			103					130	6
Snoeck et al. [67]	Luxembourg (European Region)	In the Luxembourgish population—the CON-VINCE study	Random	April–May, 2020	ELISA			911 (48.93)		Mean 47			1862	35
Sam et al. [68]	Malaysia (Western Pacific Region)	Individuals in Kuala Lumpur and Selangor, Malaysia	Random	January–June, 2020	VN			448					816	3
Pollán et al. [7]	Spain (European Region)	Spain population	Random	April–May, 2020	LFIA	Spanish: 57,858		29 349		Highest % 50–64 ≥ 65 15 094			61,075	3054
						Other: 2643								
Lundkvist et al. [69]	Sweden (European Region)	Two areas in Stockholm with different socio-economic conditions	Random	June, 2020	LFIA	Sweden as country of origin (%) 98.4		Djurgård-sstaden 42%		Mean 37			123	5
						1.1		Tensta 71%		50			90	27
Stringhini et al. [70]	Switzerland (European Region)	Former participants of the Bus Santé study and their household members	Random	April–May, 2020	ELISA			1312		Highest % 20–49 (n = 1096)		Week 1 (n = 341) 12	2766	219
												Week 2 (n = 469) 28		
												Week 3 (n = 577) 61		
												Week 4 (n = 604) 36		
												Week 5 (n = 775) 82		
Bendavid et al. [71]	USA (Region of the Americas)	Adults and children in Santa Clara County	Random	April, 2020	LFIA	Non-Hispanic 2116		1228 (36.9%)		Highest % 40–69 1706			3330	50
						White 623								
						Hispanic 266								
						Asian Other 306								
Biggs et al. [72]	USA (Region of the Americas)	The Georgia shelter-in-place order for all residents (April 3–30)	Non-random	April–May, 2020	CLIA	White, non-Hispanic 329		317		Highest % 18–49 347			696	19
						Black, non-Hispanic 266								
						Hispanic 44								
						Asian/Pacific Islander, non-Hispanic 29								
						Multiple race/ Other/ Unknown 28								
Bryan et al. [73]	USA (Region of the Americas)	Individuals in Boise, Idaho	Random	April, 2020	CLIA			2,035 (41.9)		Highest % 1,142 (23.5)			4856	87
Dietrich et al. [74]	USA (Region of the Americas)	Children in Louisiana During the State Stay at Home Order	Random	March–May, 2020	ELISA	Black 347 (42.7)		403 (49.6%)		Median 11			812	62
						White 336 (41.4)								
						Hispanic 43 (5.3)								
						Other 86 (10.6)								
Feehan et al. [38]	USA (Region of the Americas)	Individuals in New Orleans	Random	May, 2020	CLIA	White (1607)		38.2%		Mean 50.6			2640	181
						Black (828)								
						Asian (130)								
						Native American (14)								
						Multiracial /other (58)								
						Hispanic (293)								
Havers et al. [39]	USA (Region of the Americas)	Individuals in 10 Sites in the United States	Random	March–May, 2020	ELISA			7178		Highest % ≥ 65 5802			16,025	515
McLaug-hlin et al. [75]	USA (Region of the Americas)	Individuals in a Ski Resort Community, Blaine County, Idaho, US	Random	May, 2020	CLIA	Hispanic or Latino 39		438		Highest % 50 to 59 225			917	208
						Non-Hispanic or Latino 735								
Menach-emi et al. [76]	USA (Region of the Americas)	Individuals In Indiana	Random	April, 2020	CLIA	White 3373 (92)		1,656 (45)		Highest % 40–59 1,328 (36)			3658	246
						Nonwhite 281 (8)								
Ng et al. [77]	USA (Region of the Americas)	In donor and patient blood from the 2 San Francisco Bay Area	Random	March, 2020	CLIA								387	1
Rosenberg et al. [25]	USA (Region of the Americas)	Among a 15,101-patron convenience sample at 99 grocery stores in 26 counties throughout NYS	Random	April, 2020	MIA	Hispanic or Latino 17.4		47.6%		Highest % 55 + 36.1%			15,101	1887
						NH-White 58.0								
						NH-Black/African American 13.9								
						NH-Asian 8.6								
						Multiracial /Other 2.1								
Sood et al. [26]	USA (Region of the Americas)	Among adults in Los Angeles County, California	Random	April, 2020	LFIA	Hispanic 190		347		Highest % 35–54 475			863	35
						White (non-Hispanic) 497								
						Black (non-Hispanic) 72								
						Other 104								
Akinbami et al. [78]	USA (Region of the Americas)	Among healthcare, first response, and public safety personnel, Detroit metropolitan area, Michigan	Non-random	May–June 2020	ELISA	No	% Seropositive	No	% Seropositive	Highest % No	% Seropositive		16,403	1132
						Non-Hispanic White 12,858	6.0	5,146 (31.4)	6.7	45–59 5,222 (31.9)	18–24 7.9			
						Non-Hispanic Black 1,200	16.3							
						Non-Hispanic Asian 1,097	7.3							
						Hispanic 440	6.8							
						Other‡ 404	7.2							
						Declined to answer 398	7.0							
Berardis et al. [79]	Belgium (European Region)	In a Belgian cohort of patients with cystic fibrosis	Non-random	April–May 2020	CLIA			76		Mean 24.9			149	4 (2.7%)
Borges et al. [80]	Brazil (Region of the Americas)	In an asymptomatic population in Sergipe	Random	May,2020	LFIA			1469 (48.2%)		Mean 39			3046	IgM 347
														IgG 218
Borges et al. [81]	USA (Region of the Americas)	Among firefighters/paramedics of a US fire department	Non-random	April, 2020	LFIA	White 154 (78.2)		188 (93.5)		Highest % 41–50 67 (33.0)			203	18 (8.9)
						Black or African–American 9 (4.6)								
						Multi- race 8 (4.1)								
						Other 26 (13.2)								
Clarke et al. [12]	United Kingdom (European Region)	In hemodialysis patients	Non-random	April–May, 2020	CLIA	+ (129)	− (227)	+	–	+ Median	–		356	129
						Black 18	28	82 (63.6)	144 (63.4)	65	68			
						White 29	61							
						Indo-Asian 60	94							
						Other 22	44							
De Carlo et al. [82]	Italy (European Region)	In healthcare professionals of a Southern Italy hospital	Non-random	March–May,2020	CLIA					Mean 46.5		March 4	3242	62
												April 9		
												April28- May4 15		
												May 35		
Dingens et al. [83]	USA (Region of the Americas)	Among children visiting a hospital during the initial Seattle outbreak	Non-random	March–April, 2020	ELISA			541		Highest % ≥ 15 369			1076	10
Flannery et al. [84]	USA (Region of the Americas)	Among parturient women in Philadelphia	Non-random	April–June, 2020	ELISA	Black/Non-Hispanic 537		0		Median 31			1293	80
						White/Non-Hispanic 447								
						Hispanic/Latino 125								
						Asian 106								
						Other/Unknown 78								
Halatoko et al. [85]	Togo (African Region)	Among high-risk populations in Lome´ (Togo)	Random	April–May, 2020	ELISA			684 71.6%		Median 36			955	9
Hunter et al. [86]	USA (Region of the Americas)	Among healthcare workers with differing levels of coronavirus disease 2019 (Covid-19) patient exposure	Random	April–May, 2020	CLIA			30%		Mean 42.8			734	12
Khan et al. [87]	India ( South-East Asia Region)	Hospital visitors across District Srinagar	Non-random	July,2020	CLIA			1463		Highest % 30–49 1424			2906	111
Kobashi et al. [88]	Japan (Western Pacific Region)	Healthcare workers	Non-random	May,2020	CLIA			154 24.18%		Median 44			637	IgM 2
														IgG 6
Lastrucci et al. [89]	Italy (European Region)	In different essential activities during the general lock-down phase in the province of Prato (Tuscany, Italy)	Random	May,2020	ELISA			1532 (32.9%)		Median 49			4656	138 (3.0%)
Mahajan et al. [90]	USA (Region of the Americas)	Among Adults Living in Connecticut	Random	June, 2020	ELISA	Hispanic 49		244 47%		Mean 50.1			567	23 (4.1%)
						Non-Hispanic White 470								
						Non-Hispanic Black 37								
						Non-Hispanic Asian 9								
						Non-Hispanic Other 5								
Mansour et al. [91]	USA (Region of the Americas)	Among Healthcare Workers at a Tertiary Academic Hospital in New York City	Non-random	March–April, 2020	ELISA			111 (54%)		Mean 38			285	93
Mattern et al. [92]	France (European Region)	Circulation of SARS-CoV-2 in a maternity ward in an area that has been significantly affected	Non-random	May, 2020	CLIA			0		Mean 33			249	20
McDade et al. [93]	USA (Region of the Americas)	among household members of essential workers	Random	April–May, 2020	ELISA			105		Mean 37			232	30
Naranbhai et al. [94]	USA (Region of the Americas)	Chelsea residents, aged ≥ 18 years, with no current symptoms and no history of a positive SARS-CoV-2 PCR test	Non-random	April,2020	ELISA			120 (60%)		Median 46			200	63
Oliveira et al. [95]	Brazil (Region of the Americas)	In outpatients of a large public university hospital in Sao Paulo, Brazil	Random	June–August, 2020	ECLIA			156 (35.5)		Highest % 40–59			439	61
Pollán et al. [96]	Spain (European Region)	Spanish population	Random	April – May,2020	CLIA			29 349		Highest % 50–64 13 906			61,075	3054 (5%)
Psichogiou et al. [97]	Greece (European Region)	among health care workers in a country with low burden of Covid-19	Random	April- May, 2020	LFIA			453		Highest % 35–54 922			1495	15
Racine-Brzostek et al. [98]	USA (Region of the Americas)	in New York City Health Care Workers	Random	April–May, 2020	ELISA			834		Mean 37			2274	805
Shields et al. [99]	United Kingdom (European Region)	in healthcare workers	Random	April 2020	ELISA			128 (24.8%)		Median 42			516	126
Sood et al. [100]	USA (Region of the Americas)	Among adults in Los Angeles County, California	Random	April, 2020	LFIA	Hispanic 190		347		Highest % 35–54 475			863	100
						White (non-Hispanic) 497								
						Black (non-Hispanic) 72								
						Other 104								
Tang et al. [101]	China (Western Pacific Region)	In hemodialysis centers	Non-random	December, 2019- March, 2020	ELISA			619 (60.3%)		Mean 60.3			1027	47
Younas et al. [21]	Pakistan ( Eastern Mediterranean Region)	Among healthy blood donors in Karachi, Pakistan	Random	June,2020	ECLIA			380		Mean 30.6			380	128(33.6%)
Anna et al. [24]	France (European Region)	Individuals in Paris	Non-random	March–April 2020	ELISA			418 22.6%		Mean 38			1847	183
Banjar et al. [102]	Saudi Arabia ( Eastern Mediterranean Region)	Among blood donors in the early months of the pandemic in Saudi Arabia	Random	May,2020	ECLIA			796		Mean 33.3			837	12
Coatsworth et al. [103]	Australia ( Western Pacific Region)	In elective surgical patients in Australia	Non-random	June–July 2020	ELISA	White 2607 (85.8)		1479 (48.7)		Mean 54			3037	15
						Asian 203 (6.7)								
						ATSI 16 (0.5)								
						Black/African 19 (0.6)								
						Other 192 (6.3)								
Ebinger et al. [104]	USA (Region of the Americas)	In healthcare workers	Random	May,2020	CLIA	(−) Asian 1809 (31)	( +) 57 (27)	1876 (32)	73 (34)	Mean (-) 41.6	Mean ( +) 38.5		6062	212
						Black 354 (6)	18 (8)							
						White 2938 (50)	104 (49)							
						Other 749 (13)	33 (16)							
Kantele et al. [105]	Finland (European Region)	Among healthcare workers at Helsinki University Hospital, Finland	Non-random	March–April 2020	ELISA			187 (17.3%)		Median 38			1095	33
Ladoire et al. [106]	France (European Region)	Among the staff and patients of a French cancer center after first lockdown	Non-random	May–June 2020	ECLIA			Employees ( +) 2 (16.7%)	(−) 139 (21.4%) 649	Mean ( +) 35.3	38.6		663	12
								Patients ( +) 7 (41.2%)	299 (30.1%)	Mean ( +) 65.2	63.1		1011	17
Laursen et al. [107]	Sweden- Denmark (European Region)	Among Danish and Swedish Falck Emergency and Non-Emergency Healthcare Workers	Random	June–August 2020	LFIA	Swedish 1248		1939 (59.3)		Highest % 40–60 1732 (52.9)			3272	159 (4.9%)
						Danish 2024								
Lombardi et al. [108]	Italy (European Region)	Among healthcare workers of a large university hospital in Milan, Lombardy, Italy	Random	April–June 2020	CLIA			1232		Mean 44.8			4055	309
Moncunill et al. [109]	Spain (European Region)	Among health care workers in a Spanish hospital after 3 months of follow-up	Random	April–May 2020	ELISA			206		Mean 42			565	82
Pan et al. [110]	Taiwan ( Western Pacific Region)	Among healthcare workers in a tertiary care hospital in Taiwan	Random	July–Aug 2020	ELISA			70 36.8%		Mean 36.3			194	64
Pereckait et al. [111]	Lithuania (European Region)	In healthcare workers of Kaunas Hospitals	Random	June–September 2020	LFIA			63		Mean 43.4			432	5
McQuade et al. [112]	USA (Region of the Americas)	Among Outpatients in Virginia	Random	June–August, 2020	ELISA	Hispanic 396		1556 (33.3)		Mean 48.8			4675	101
						Non-Hispanic 4279								
Venugopal et al. [113]	USA (Region of the Americas)	Among health care workers in a New York City hospital	Random	March–May, 2020	ELISA	Hispanic 132 (28%)		149 (31%)		Highest % 20–39 230			478	130
						Black 87 (18%)								
						Asian 114 (24%)								
						Other race 30 (6%)								
						Caucasian 115 (24%)								
Malagón- Rojas et al. [114]	Colombia (Region of the Americas)	Healthcare workers in Colombia	Random	September–November 2020	CLIA	Afro- Colombian 216		788		36.45 ± 10.5			3296	1021
						White 995								
						Indigenous 112								
						Mestizo 2004								
						Raizal 19								
						Gipsy 6								
Poustchi et al. [115]	Iran (Eastern Mediter-ranean Region)	High-risk occupational groups	Random	April 17 and June 2, 2020	ELISA			1795		Highest % 30–39 2995(33·6%)			3530	494
Poulikakos et al. [116]	England (European Region)	Healthcare workers in a tertiary center in North West	Random	May 2020	ELISA	Black or BAME 55 (19·6%)		205 (73%)					281	17
						did not declare ethnicity 25 (8·9%)								
						DIPC 195 (69·4%)								
Amendola et al. [117]	Italy (European Region)	Healthcare workers of the largest children hospital in Milan	Non-random	April 15, 2020	ELISA			108		Median 44			663	34
Brandstetter et al. [118]	Germany (European Region)	Hospital staff	Random	March 2020	ELISA			30		Highest % 36–50 72 (35.8)			201	31
Chibwana et al. [119]	Malawi (African Region)	Health Care Workers	Random	May 2020 to June 2020	ELISA			236		Median 31			500	84

The quality assessment checklist of the observational studies showed that most of these studies had a good quality. Except for a few of the studies had unknown parts in the checklist (Table 2).

**Table 2:** Results of quality assessment based on JBI checklist

	Inclusion criteria	Detailed description of the population	Exposure (validity and reliability)	Condition	Identification of confounding factors	Deal with confounding factors	Outcome	Statistical analysis
Herzog et al.	Yes	Yes	Yes	Yes	Unclear	Unclear	Yes	Yes
Filho et al.	Yes	Yes	Yes	Yes	Yes	Yes	Yes	Yes
Silveira et al.	Yes	Yes	Yes	Yes	Yes	Yes	Yes	Yes
Torres et al.	Yes	Yes	Yes	Yes	Yes	Yes	Yes	Yes
Chang et al.	Yes	Yes	Yes	Yes	Yes	Yes	Yes	Yes
To et al.	Yes	Yes	Yes	Yes	Yes	Yes	Yes	Yes
Liang et al.	Yes	Yes	Yes	Yes	Yes	Unclear	Yes	Unclear
Jerković et al.	Yes	Yes	Yes	Yes	Yes	Yes	Yes	Yes
Erikstrup et al.	Yes	Yes	Yes	Yes	Yes	Yes	Yes	Yes
Petersen et al.	Yes	Yes	Yes	Yes	Yes	Unclear	Yes	Yes
Ward et al.	Yes	Yes	Yes	Yes	Yes	Yes	Yes	Unclear
Gallian et al.	Yes	Yes	Yes	Yes	Yes	Unclear	Yes	Unclear
Grzelak et al.	Yes	Yes	Yes	Yes	Yes	Yes	Yes	Yes
Fischer et al.	Yes	No	Yes	Yes	Yes	Unclear	Yes	Unclear
Weis et al.	Yes	Yes	Yes	Yes	Yes	Yes	Yes	Yes
Bogogiannidou et al.	No	Yes	Yes	Yes	Yes	Unclear	Yes	Yes
Merkely et al.	Yes	Yes	Yes	Yes	Yes	Yes	Unclear	Yes
Shakiba et al.	Yes	Yes	Yes	Yes	Yes	Unclear	Yes	Yes
Percivalle et al.	Yes	Yes	Yes	Yes	Unclear	Unclear	Yes	No
Valenti et al.	Yes	Yes	Yes	Yes	Yes	Yes	Yes	Yes
Fiore et al.	Yes	No	Yes	Yes	Yes	Unclear	Yes	Yes
Doi et al.	Yes	Yes	Yes	Yes	Yes	Yes	Yes	Unclear
Takita et al.	Yes	Yes	Yes	Yes	Yes	Yes	No	Yes
Takita et al.	Yes	Yes	Yes	Unclear	Yes	Yes	Yes	Unclear
Uyoga et al.	Yes	Yes	Yes	Yes	Yes	Yes	Unclear	Unclear
Song et al.	Yes	Yes	Yes	Yes	Yes	Yes	Yes	Yes
Kammon et al.	Yes	Yes	Yes	Yes	Yes	Yes	Yes	Unclear
Snoeck et al.	Yes	Yes	Yes	Yes	Yes	Unclear	Yes	Yes
Sam et al.	Yes	No	Yes	Yes	Yes	Unclear	Yes	Unclear
Pollán et al.	Yes	Yes	Yes	Yes	Unclear	Unclear	Yes	Yes
Lundkvist et al.	Yes	Yes	Yes	Yes	Yes	Unclear	Yes	Unclear
Stringhini et al.	Yes	Yes	Yes	Yes	Yes	Yes	Yes	Yes
Bendavid et al.	Yes	Yes	Yes	Yes	Yes	Yes	Yes	Yes
Biggs et al.	Yes	Yes	Yes	Yes	Yes	Unclear	Yes	Unclear
Bryan et al.	Yes	Yes	Yes	Yes	Yes	Yes	Yes	Unclear
Dietrich et al.	Yes	Yes	Yes	Yes	Yes	Yes	Unclear	Yes
Feehan et al.	Yes	Yes	Yes	Yes	Yes	Yes	Yes	Unclear
Havers et al.	Yes	Yes	Yes	Yes	Yes	Yes	Yes	Unclear
McLaughlin et al.	Yes	Yes	Yes	Yes	Yes	Unclear	Unclear	No
Menachemi et al.	Yes	Yes	Yes	Yes	Yes	Unclear	Yes	Unclear
Ng et al.	Yes	No	Yes	Yes	Yes	Unclear	Yes	Yes
Rosenberg et al.	Yes	Yes	Yes	Yes	Yes	Yes	Yes	Yes
Sood et al.	Yes	Yes	Yes	Yes	Yes	Yes	Yes	Unclear
Akinbami et al.	Yes	Yes	Yes	Yes	Yes	Yes	Yes	Yes
Berardis et al.	Yes	Yes	Yes	Yes	Yes	Yes	Unclear	No
Borges et al.	Yes	Yes	Yes	Yes	Yes	Yes	Yes	Yes
Caban-Martinez et al.	Yes	Yes	Yes	Yes	Yes	Yes	No	Yes
Clarke et al.	Yes	Yes	Yes	Yes	Yes	Yes	Yes	Yes
De Carlo et al.	Yes	Yes	Yes	Yes	Yes	Unclear	Yes	Yes
Dingens et al.	Yes	Yes	Yes	Yes	Yes	Yes	Yes	Yes
Flannery et al.	Yes	Yes	Yes	Yes	Unclear	Unclear	Yes	Yes
Halatoko et al.	Yes	Yes	Yes	Yes	Yes	Yes	Yes	Yes
Hunter et al.	Yes	Yes	Yes	Yes	Yes	Yes	Yes	Yes
Khan et al.	Yes	Yes	Yes	Yes	Yes	Yes	Yes	Yes
Kobashi et al.	Yes	Yes	Yes	Yes	Yes	Yes	Yes	Unclear
Lastrucci et al.	Yes	Yes	Yes	Yes	Yes	Yes	Yes	Yes
Mahajan et al.	Yes	Yes	Yes	Yes	Yes	Unclear	Yes	Yes
Mansour et al.	Yes	Yes	Yes	Yes	Yes	Unclear	Yes	Yes
Mattern et al.	Yes	Yes	Yes	Yes	Yes	Unclear	No	Yes
McDade et al.	Yes	Yes	Yes	Yes	Yes	Yes	Unclear	Yes
Naranbhai et al.	Yes	Yes	Yes	Yes	Yes	Unclear	Yes	Yes
Oliveira et al.	Yes	Yes	Yes	Yes	Unclear	Unclear	Yes	Yes
Psichogiou et al.	Yes	Yes	Yes	Yes	Yes	Yes	Yes	Yes
Racine-Brzostek et al.	Yes	Yes	Yes	Yes	Yes	Yes	Yes	No
Shields et al.	Yes	Yes	Yes	Yes	Yes	Unclear	Yes	Yes
Sood et al.	Yes	Yes	Yes	Yes	Yes	Yes	Yes	Unclear
Tang et al.	Yes	Yes	Yes	Yes	Yes	Yes	Yes	Yes
Younas et al.	Yes	No	Yes	Yes	Yes	Yes	Unclear	Yes
Anna et al.	Unclear	Yes	Yes	Yes	Yes	Unclear	Yes	Yes
Banjar et al.	Yes	Yes	Yes	Yes	Unclear	Unclear	Yes	Yes
Coatsworth et al.	Yes	Yes	Yes	Yes	Yes	Yes	Yes	Unclear
Ebinger et al.	Yes	Yes	Yes	Yes	Yes	Yes	Yes	Yes
Kantele et al.	Yes	Yes	Yes	Yes	Yes	Yes	Yes	Yes
Ladoire et al.	Yes	Yes	Yes	Yes	Yes	Yes	Yes	Yes
Laursen et al.	Yes	Yes	Yes	Yes	Yes	Yes	Yes	Unclear
Lombardi et al.	Yes	Yes	Yes	Yes	Yes	Unclear	Yes	Yes
Moncunill et al.	Yes	Yes	Yes	Yes	Yes	Yes	No	Yes
Pan et al.	Yes	Yes	Yes	Yes	Yes	Unclear	Yes	Unclear
Pereckait et al.	Yes	Yes	Yes	Yes	Unclear	Unclear	Yes	Unclear
McQuade et al.	Yes	Yes	Yes	Yes	Yes	Yes	No	Yes
Venugopal et al.	Yes	Yes	Yes	Yes	Yes	Yes	Yes	Yes
Malagón- Rojas et al.	Yes	Yes	Yes	Yes	Yes	Unclear	Yes	Yes
Poustchi et al.	Yes	Yes	Unclear	Yes	Yes	Yes	Yes	Yes
Poulikakos et al.	Yes	Yes	Yes	Yes	No	Yes	No	Unclear
Amendola et al.	Yes	Yes	Yes	Yes	Yes	Yes	Yes	Yes
Brandstetter et al.	Yes	Yes	Yes	Yes	Yes	Unclear	Yes	Unclear
Chibwana et al.	Yes	Yes	Yes	Yes	Yes	Yes	Yes	Yes

### Seropositive in Eastern Mediterranean population

**Fig. 2:**
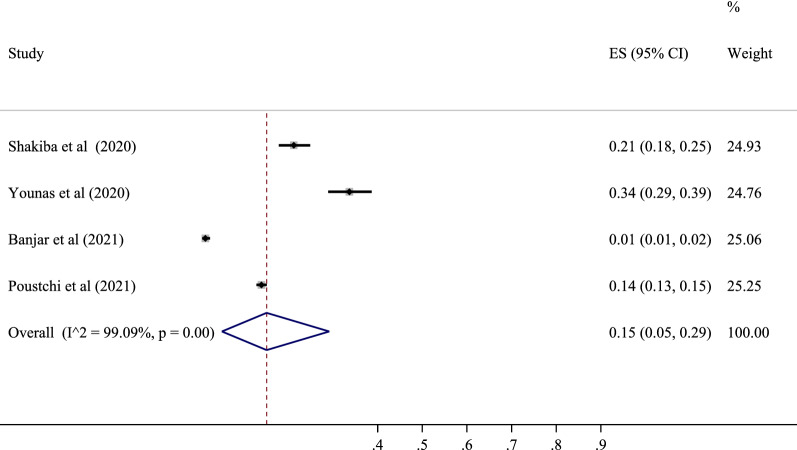
The pooled prevalence of SARS-CoV-2 seropositive in Eastern Mediterranean population

Four studies with a total sample size of 5298 cases determined the prevalence of SARS-CoV-2 in this area. The lowest correlation belonged to the study of Banjar et al. with a prevalence of 1% (95% CI 1 to 2%), and the highest prevalence belonged to the study of Younas et al. with a prevalence of 34% (95% CI 29 to 39%). After combining the results of these studies, the pooled estimate was equal to 15%, with a 95% confidence interval of 5 to 29% (Figs. 2 and 7). The highest value was in Pakistan with a prevalence of 24% (95% CI 19 to 39%), and the lowest was in Saudi Arabia with a prevalence of 1% (95% CI 1 to 2%) (Table 3).

**Table 3:** The subgroup analysis related to region; the prevalence was examined based on the Courtiers

Regions	Courtiers	Pooled prevalence (95% CI)	Heterogeneity assessment	
			I square	P _heterogeneity_
America	Overall	8% (6–11%)	99.54%	0.000
	Brazil	7% (2–12%)	90.39%	0.000
	USA	9% (7–11%)	93.66%	0.000
	Chile	11% (9–13%)	–	–
	Colombia	29% (31–23%)	–	–
European	Overall	5% (4–6%)	98.99%	0.000
	Belgium	5% (3–8%)	–	–
	Croatia	1% (0–3%)	–	–
	Denmark	2% (1–4%)	68.65%	0.040
	England	20% (4–45%)	76.00%	0.021
	Finland	3% (2–4%)	–	–
	France	4% (1–9%)	87.08%	0.000
	Germany	7% (0–19%)	88.68%	0.000
	Greece	1% (0–2%)	–	–
	Italy	5% (3–9%)	86.58%	0.000
	Spain	6% (5–7%)	74.04%	0.001
	Sweden	5% (4–6%)	88.00%	0.000
	Switzerland	8% (5–10%)	–	–
	Hungary	1% (1–2%)	–	–
	Finland	3% (2–4%)	–	–
	Croatia	1% (1–2%)	–	–
	Luxemburg	2% (1–3%)	–	–
	Lithuania	1% (0–3%)	–	–
Western Pacific	Overall	3% (2–4%)	96.82%	0.000
	China	2% (1–3%)	89.91%	0.000
	Japan	3% (1–5%)	87.82%	0.001
	Australia	0% (0–2%)	–	–
	Korea	8% (4–12%)	55.84%	0.094
	Malaysia	0% (0–2%)	–	–
	Taiwan	33% (26–40%)	–	–
	India	4% (2–6%)	–	–
Eastern Mediterranean	Overall	15% (5–29%)	99.09%	0.000
	Iran	15% (12–17%)	–	–
	Pakistan	24% (19–39%)	–	–
	Saudi Arabia	1% (1–2%)	–	–
Africa	Overall	6% (1–13%)	97.87%	0.000
	Libya	5% (2–10%)	–	–
	Kenya	6% (5–6%)	–	–
	Togo	1% (0–2%)	–	–
	Malavi	17% (14–20%)	–	–

### Seropositive in Africa population

**Fig. 3:**
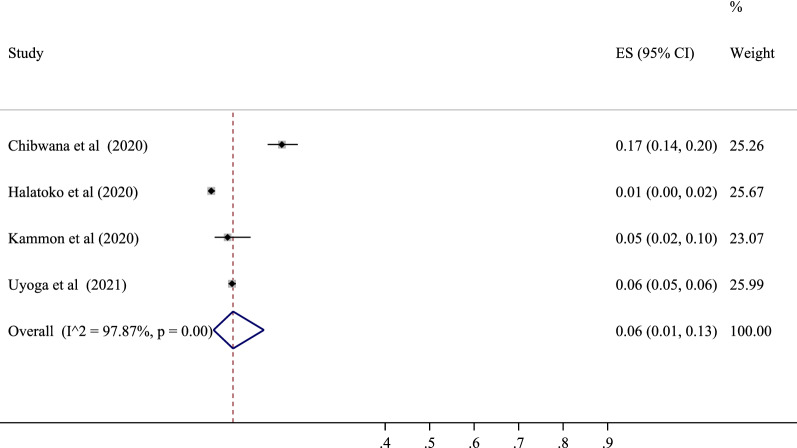
The pooled prevalence of SARS-CoV-2 seropositive in Africa population

Four studies were performed to determine the prevalence of SARS-CoV-2 positive serological tests in this area. The lowest correlation belonged to the study of Halatoko et al. with a prevalence of 1% (95% CI 0 to 2%), and the highest prevalence belonged to the study of Chibwana et al. with a prevalence of 17% (95% CI 14 to 20%). After combining the results of these studies, the pooled estimate was equal to 6%, with a 95% confidence interval of 1 to 13% (Figs. 3 and 7). Also, among the countries in this region, the highest value was related to Malawi with a prevalence of 17% (95% CI 14 to 20%) and the lowest to Togo with a prevalence of 1% (95% CI 0 to 2%) (Table 3).

### Seropositive in America population

**Fig. 4:**
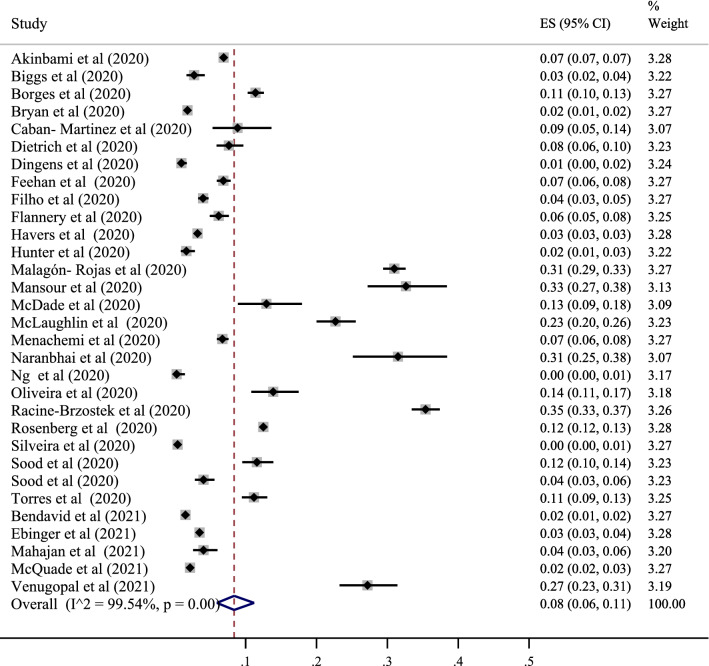
The pooled prevalence of SARS-CoV-2 seropositive in America population

Thirty-one studies determined the prevalence of SARS-CoV-2 positive serological tests in this area, with the lowest correlation belonging to the study of Ng et al. with a prevalence of 0% (95% CI 0 to 1%) and also the study of Silveira et al. with a prevalence of 0% (95% CI 0 to 1%). The highest prevalence belonged to the study of Racine-Brzostek et al., with a prevalence of 35% (95% CI 33 to 37%). After combining the results of these studies, the pooled estimate was equal to 8%, with a 95% confidence interval of 6 to 10% (Figs. 4 and 7). According to the analysis, among the countries in this region, the highest value was related to Colombia with a prevalence of 29% (95% CI 23 to 31%) and the lowest to Brazil with a prevalence of 7% (95% CI 2 to 12). %) (Table 3).

In the subgroup analysis related to this area, the prevalence was also examined based on the population type (healthy and unhealthy), the diagnostic test type (ELISA–CLISA–LFIA), the sampling type (random and non-random), time (months after pandemic), the perspective (local–regional–national), and the type of the study (cohort–cross-sectional). According to the classification based on the type of population, the results showed that the serological test's positivity was 5% in healthy people (95% CI 4 to 6%). In addition, the evaluation results differed according to the test type, and the prevalence of positive tests was 12% for ELISA (95% CI 10 to 15%), 6% for CLISA (95% CI 4 to 8), and 6% for LFIA (95% CI 4 to 9%). The results showed that the highest prevalence occurred in the diagnostic subgroup of ELISA. Also, depending on the type of sampling, in randomized studies, the prevalence was 9% (95% CI 7 to 11%), and in non-randomized studies, the prevalence was 10% (95% CI 7 to 13%). This indicated a higher prevalence in the non-randomized group. Based on the months after pandemic, the prevalence were 7% for 4 month (95% CI 3 to 12%), 8% for 5 month (95% CI 5 to 13%), 9% for 6 month (95% CI 6 to 14%), and 11% for 7 month (95% CI 0 to 32%). Over time, this prevalence increased. Prevalence based on perspective was 12% for local (95% CI 6 to 19%), 6% for regional (95% CI 4 to 10%), and 3% for national (95% CI 4 to 10%), which was higher in local studies. Also, prevalence was 7% for cohort (95% CI 2 to 14%), and 9% for cross-sectional (95% CI 6 to 12%). Prevalence was higher in cross-sectional studies (Table 4).

**Table 4:** The subgroup analysis related to region, the prevalence was examined based on the population type (healthy and unhealthy), the diagnostic test type (ELISA–CLISA–LFIA–VN), and the sampling type (random and non-random)

Regions	Variables		Pooled prevalence (95% CI)	Heterogeneity assessment	
				I square	P _heterogeneity_
Western Pacific	Study population	Healthy	3% (2–5%)	90.20%	0.000
		Un-healthy	2% (1–3%)	91.55%	0.000
	Diagnostic methods	ELISA	7% (3–10%)	17.03%	0.281
		CLIA	1% (0–2%)	0.00%	0.320
		LFIA	4% (3–5%)	41.35%	0.160
		VN	1% (0–2%)	55.02%	0.301
	Sampling methods	Random	4% (2–5%)	89.65%	0.000
		Non-random	2% (0–4%)	84.23%	0.000
	Time	2 months after pandemic	2% (1–3%)	93.20%	0.000
		4 months after pandemic	3% (2–5%)	–	–
		5 months after pandemic	4% (3–5%)	–	–
		6 months after pandemic	2% (1–3%)	–	–
		7 months after pandemic	1% (1–2%)	–	–
		8 months after pandemic	5% (4–6%)	–	–
	Perspective	Local	4% (2–6%)	91.05%	0.000
		Regional	3% (1–5%)	89.04%	0.000
		National	–	–	–
	Type of study	Cohort	2% (1–3%)	88.08%	0.000
		Cross-sectional	4% (2–6%)	91.90%	0.000
European	Study population	Healthy	5% (4–6%)	92.15%	0.000
		Un-healthy	20% (16–23%)	89.22%	0.000
	Diagnostic methods	ELISA	6% (4–8%)	78.65%	0.030
		CLIA	6% (3–9%)	79.99%	0.001
		LFIA	4% (2–8%)	90.36%	0.000
		VN	7% (5–8%)	77.00%	0.000
		ECLIA	1% (1–3%)	–	
	Sampling methods	Random	5% (4–6%)	97.68%	0.000
		Non-random	6% (3–8%)	90.22%	0.000
	Time	2 months after pandemic	23% (19–28%)	88.17%	0.000
		3 months after pandemic	5% (4–7%)	89.08%	0.000
		4 months after pandemic	4% (2–7%)	92.54%	0.000
		5 months after pandemic	6% (5–8%)	84.28%	0.000
		6 months after pandemic	3% (2–6%)	98.90%	0.000
		7 months after pandemic	5% (3–7%)	87.09%	0.000
	Perspective	Local	8% (6–11%)	89.00%	0.000
		Regional	6% (3–8%)	88.89%	0.000
		National	3% (2–4%)	83.49%	0.000
	Type of study	Cohort	5% (2–8%)	99.90%	0.000
		Cross-sectional	6% (5–7%)	98.56%	0.000
America	Study population	Healthy	9% (8–12%)	92.19%	0.000
		Un-healthy	–	–	–
	Diagnostic methods	ELISA	12% (10–15%)	79.00%	0.001
		CLIA	6% (4–8%)	81.54%	0.001
		LFIA	6% (4–9%)	88.99%	0.000
		VN	–	–	–
	Sampling methods	Random	9% (7–11%)	97.22%	0.000
		Non-random	10% (7–13%)	98.48%	0.000
	Time	4 months after pandemic	7% (3–12%)	89.22%	0.000
		5 months after pandemic	8% (5–13%)	80.29%	0.000
		6 months after pandemic	9% (6–14%)	93.00%	0.000
		7 months after pandemic	11% (0–32%)	92.33%	0.000
	Perspective	Local	12% (6–19%)	99.52%	0.000
		Regional	6% (4–10%)	92.54%	0.000
		National	3% (4–10%)	–	–
	Type of study	Cohort	7% (2–14%)	79.90%	0.000
		Cross-sectional	9% (6–12%)	77.56%	0.000

### Seropositive in European population

**Fig. 5:**
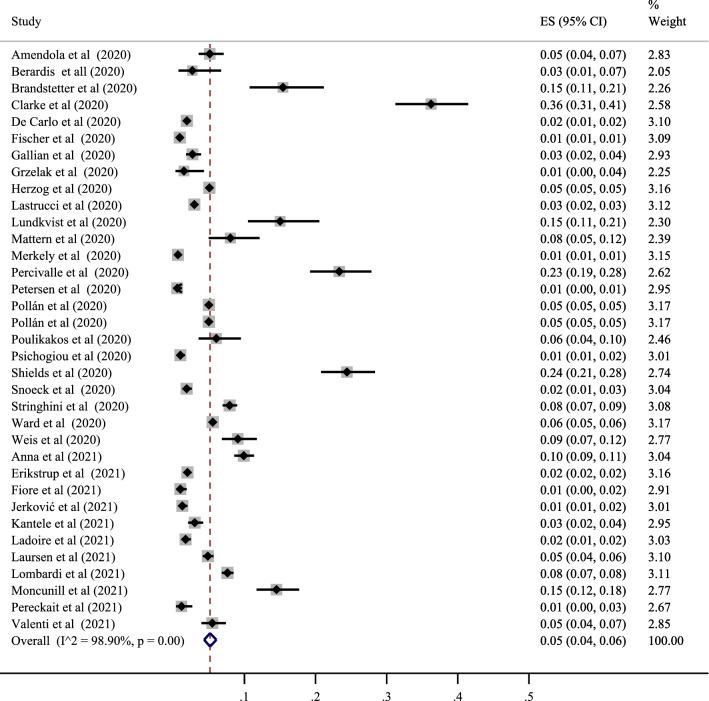
The pooled prevalence of SARS-CoV-2 seropositive in European population

In addition, 35 studies determined the prevalence of SARS-CoV-2 positive serological tests in this area with the lowest correlation belonging to the study of Fischer et al. with a prevalence of 01% (95% CI 01 to 01%) and also the study of Merkely et al. with a prevalence of 01% (95% CI 01 to 011%). The highest correlation belonged to the study of Clarke et al., with a prevalence of 36% (95% CI 31 to 41%). After combining the results of these studies, the pooled estimate was equal to 5% with a 95% confidence interval of 4 to 6% (Figs. 5 and 7). In addition, the highest value was related to the United Kingdom among the countries in this region, with a prevalence of 20% (95% CI 4 to 45%). The lowest was associated with Greece, with a prevalence of 1% (95% CI 0 to 2%) (Table 3).

In the subgroup analysis related to this area, the prevalence was also examined based on the population type (healthy and unhealthy), the diagnostic test type (ELISA–CLISA–LFIA–VN–ECLIA), and the sampling type (random and non-random), time (months after pandemic), the perspective (local–regional–national), and the type of the study (cohort–cross-sectional). The classification results by the population type showed the positivity of the serological test in the healthy and unhealthy populations at 5% (95% CI 4 to 6%) and 20% (95% CI 16 to 23%), respectively. Prevalence in the unhealthy population was higher. The results obtained based on the type of the diagnostic test were different, and the prevalence of positive tests was 6% for ELISA (95% CI 4 to 8%), 6% for CLISA (95% CI 3 to 9%), 4% for LFIA (95% CI 2 to 8%), 7% for VN (95% CI 5 to 8%), and 1% for ECLIA (95% CI 1 to 3%). The highest value was evaluated in VN type. Also, depending on the type of sampling, the prevalence in randomized studies was 5% (95% CI 4 to 6%), and in non-randomized studies, it was 6% (95% CI 3 to 8%). Prevalence was higher in non-randomized studies (Table 4). For the months after pandemic, the prevalence were 23% for 2 month (95% CI 19 to 28%), 5% for 3 month (95% CI 4 to 7%), 4% for 4 month (95% CI 2 to 7%), 6% for 5 month (95% CI 5 to 8%), 3% for 6 month (95% CI 2 to 6%), and 5% for 7 month (95% CI 3 to 7%).The highest prevalence was in the 2 months after the pandemic. Prevalence based on perspective was 8% for local (95% CI 6 to 11%), 6% for regional (95% CI 3 to 8%), and 3% for national (95% CI 2 to 4%) indicating higher prevalence in local studies. Prevalence based on type of study was 5% for cohort (95% CI 2 to 8%), and 6% for cross-sectional (95% CI 5 to 7%). Prevalence was higher in cross-sectional studies (Table 4).

### Seropositive in Western Pacific population

**Fig. 6:**
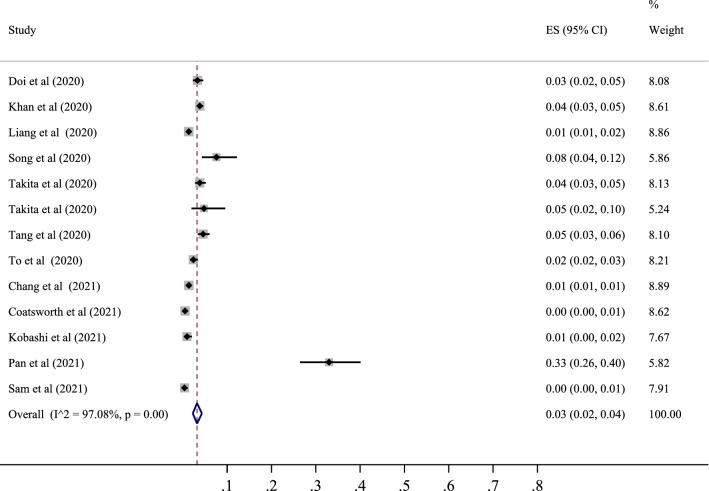
The pooled prevalence of SARS-CoV-2 seropositive in Western Pacific population

**Fig. 7:**
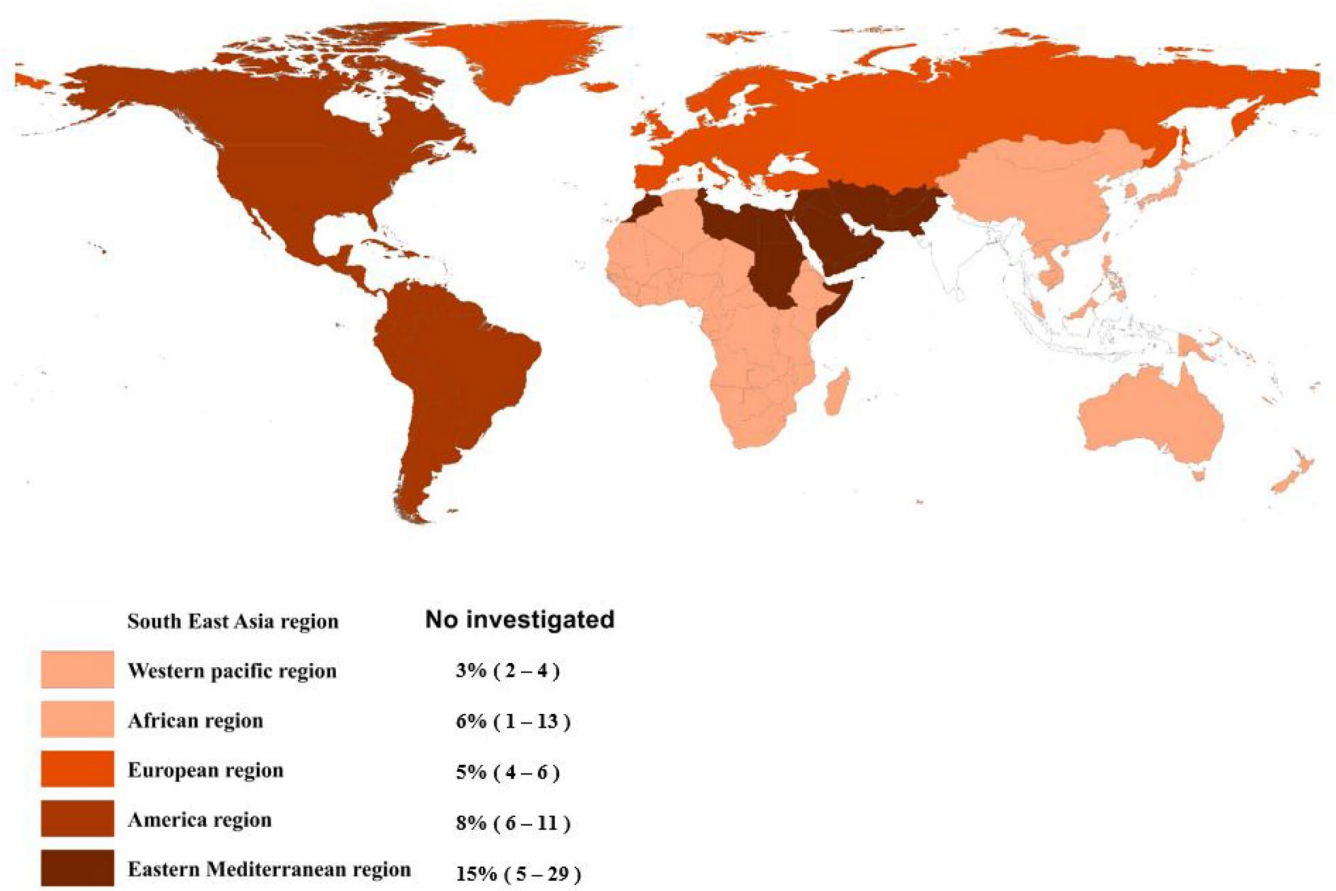
Seroprevalence rates of SARS-CoV-2 in the general human population in different countries using the geographic information system (GIS)

Finally, 12 studies determined the prevalence of SARS-CoV-2 positive serological tests in this area, with the lowest correlation belonging to the study of Coatsworth et al. with a prevalence of 0% (95% CI 0 to 1%) and the highest correlation belonging to the study of Pan et al. with a prevalence of 33% (95% CI 27 to 40%). After combining the results of these studies, the pooled estimate was equal to 3%, with a 95% confidence interval of 2 to 4% (Figs. 6 and 7). Finally, among the countries in this region, the highest value was related to Taiwan with a prevalence of 33% (95% CI 23 to 40%), and the lowest was associated with Malaysia with a prevalence of 0% (95% CI 0 to 2%) (Table 3).

In the subgroup analysis related to this region, the prevalence was also examined based on the population type (healthy and unhealthy), the diagnostic test type (ELISA–CLISA–LFIA–VN), and the sampling type (random and non-random). The classification results based on the population type showed that the serological test was positive in 3% of the healthy population (95% CI 2 to 5%) and 2% of the unhealthy population (95% CI 1 to 3%). It was higher in the healthy population than in the unhealthy one. The results obtained based on the type of diagnostic test were different. The prevalence of positive tests was 7% for ELISA (95% CI 3 to 10%), 1% for CLISA (95% CI 0 to 2%), 4% for LFIA (95% CI 3 to 5%) and 1% for VN (95% CI 0 to 2%). The highest value was observed in the ELISA group. Also, depending on the type of sampling, the prevalence was 4% in randomized studies (95% CI 2 to 5%), and in non-randomized studies, the prevalence was 2% (95% CI 0 to 4%). The prevalence in the randomized group was higher than that in the non-randomized one (Table 4).

### Meta-regression results

**Fig. 8:**
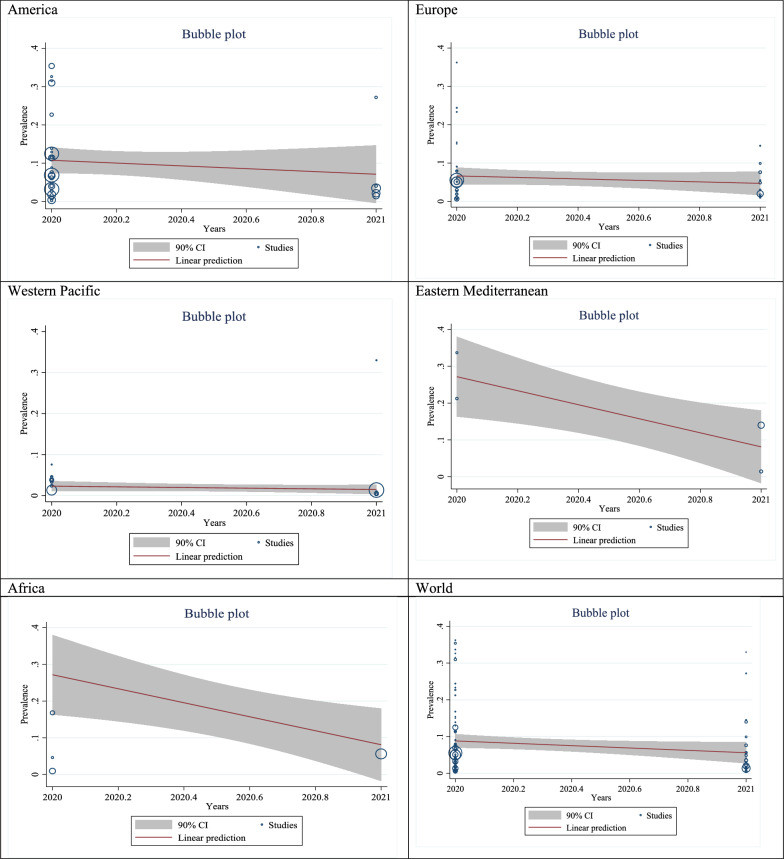
Meta-regression analysis of estimated pooled prevalence in WHO regions and around the world from 2020 to 2021. America (B: − 0.03, SE: 0.05, P: 0.469). Europe (B: − 0.01, SE: 0.02, P: 0.401). Western Pacific (B: − 0.01, SE: 0.01, P: 0.430). Eastern Mediterranean (B: − 0.19, SE: 0.08, P: 0.033). Africa (B: 0.01, SE: 0.02, P: 0.854). World (B: − 0.03, SE: 0.02, P: 0.122)

In this part, we analyzed the changes in SARS-CoV-2 seroprevalence in different WHO regions and worldwide based on the year from 2020 to 2021. The result in America (B: − 0.03, SE: 0.05, P: 0.469), Europe (B: − 0.01, SE: 0.02, P: 0.401), Western Pacific (B: − 0.01, SE: 0.01, P: 0.430), Eastern Mediterranean (B: − 0.19, SE: 0.08, P: 0.033) and around the World (B: − 0.03, SE: 0.02, P: 0.122) was decreasing which in Western Pacific and World was significant. However, the result in Africa (B: 0.01, SE: 0.02, P: 0.854) was increased (Fig. 8).

## Discussion

Due to the current Covid-19 pandemic, the prevalence and incidence of this disease are increasing worldwide. Because antibodies are produced in response to many pathogens, including Covid-19, and have a higher advantage than other diagnostic methods in determining the serology prevalence, here we have globally collected verified data (by September 2020) to contribute to a comprehensive understanding of the current pandemic by conducting a comprehensive review of the prevalence of Covid-19 serology in different populations and geographical areas. In this meta-analysis, the cumulative prevalence was calculated at 414,773 based on the studied research, and 25,065 people in the world were infected with Covid-19 by the date of this study.

The results obtained based on the study region showed that among the six regions of the WHO, Eastern Mediterranean and Western Pacific had the highest (15%) and lowest (3%) prevalence, respectively. The largest sample size and number of studies were related to the European Region, accompanied by other development characteristics in this region. It is also impossible to accurately assess the Covid-19 prevalence based on just one study at the local level. Still, one can imagine the general situation from these few studies, especially globally. Although the exact protective effect of antibodies against mutant variants has not been determined so far [21], it can be said that the differences observed in seroprevalence are probably related to differences in the disease transmission status in the community due to behavioral differences, the public health status, local resources, and environmental issues. Of course, there are other issues, such as altitude and climatic differences, and the relevant evidence is not yet complete [22, 23]. Differences in the volume, time, single approach, sampling method, missing samples, sample size, selection bias, greater participation of symptomatic individuals, the inclusion of minority populations, lack of validity and reliability of questionnaires in determining symptoms, accuracy of diagnostic kits, rate of decrease in the antibody titer, possible reinfection, the persistence of the virus in a large population of the society, and diversity of geographical and demographic characteristics (age, sex, race, ethnicity, etc.) were among the limiting factors in most studies [24–26].

In the present study, the lowest Covid-19 seroprevalence was in Western Pacific and African countries, followed by European and American ones, and was slightly higher in the Eastern Mediterranean. However, within each of the World Health Organization's geographical areas, there were significant differences. For example, the estimated prevalence in Taiwan (33%) was much higher than that of other Western Pacific countries. The same difference existed in Europe, so the United Kingdom, with an estimated prevalence of 20%, was significantly different from its neighbors. In contrast, the differences in the Americas and Africa were relatively small, and the Covid-19 seroprevalence was moderate in these regions. Finally, in the Eastern Mediterranean region, Covid-19 seroprevalence was relatively high in Iran and Pakistan, except in Saudi Arabia. Similar studies that have mainly classified the prevalence based on countries' income reported that in some cases, middle-income countries and, in other instances, high-income countries had reported a higher prevalence [27, 28]. So, we could not find a precise correlation between the income level of countries and the Covid-19 seroprevalence, which may be due to differences in the time of epidemic changes in these countries, sampling and laboratory methods, disease control policies, and vaccination in different populations.

Studies used different serological tests. Due to the many reasons presented for the difference in Covid-19 seroprevalence in additional studies and populations, it was impossible to precisely determine the effect of the test type on this rate. Various studies showed that the type of used antigen, the number of passed days since the onset of the patient’s initial symptoms, and the performance of the serological test itself affected the sensitivity and specificity of various tests [29–31]. The reported sensitivity for different tests was from 66 to 97%, while the specificity of all tests was reported to be higher than 95% [32, 33].

Different demographic subgroups such as healthy and unhealthy individuals and the randomized and non-randomized sampling, in general, can affect the difference in seroprevalence. As stated in the present study, studies reported lower and higher seroprevalence in different geographic perspectives and time from the beginning of the pandemic areas in each category. For example, in the Western Pacific countries, the seroprevalence of healthy populations was higher than that of unhealthy ones. In cases with the random sampling method, it was more than the non-random one. Also, in our study, the seroprevalence increased from local to national perspectives, respectively, due to the impact of more facilities, effective health policies, and easier access to health care services at the national level. In general, the samples taken in our study were in the time period from 2 January to 21 September 2020. In this period, clinical management of the disease was based on symptomatic therapies. Still, non-pharmaceutical interventions (NPIs) such as physical distance in all settings, hand hygiene and use of protective equipment self and large-scale isolation, and closure of borders, schools, and workplaces play a critical role in preventing and controlling disease transmission. Therefore, problems with infrastructure, imports of some drugs, and strategies such as quarantine, proper promotion, or non-observance of the mentioned factors can change the prevalence of the disease months from the beginning of the pandemic. For example, the prevalence peaked in Western Pacific and European countries in April 2020.

Also, specific mutations in the SARS-CoV-2 genome over time impacted diagnostics, transmissibility, and treatment. And the first variant (alpha) was identified in late 2020, so the obtained seroprevalence pattern cannot be justified by Covid-19 variants [34, 35]. Hence, there were no effective and available vaccines or drugs against Covid-19 in our study period. The first public vaccine was given to a 91-year-old woman in The UK named Margaret Keenan on 8th December 2020 [36]; the results of the current meta-analysis may be less justified by vaccination and viral variants, so conducting such seroprevalence studies would need to be done again carefully.

In the meta-regression performed based on the observed changes in Covid-19 seroprevalence over time, it was found that other countries showed a downward trend despite our expectation of this increase over time, except in the subgroup of African countries in Covid-19 seroprevalence. This may be due to differences in sampling times in different countries due to the peak of the disease and changes in prevention systems in these countries on the one hand and the instability of Covid-19 specific antigens over time on the other hand.

One of the strengths of this study was the global review of Covid-19 seroprevalence studies. Also, in this research, studies were aggregated by different regions of the World Health Organization, while in similar studies, classification was more based on the income level of countries [27, 28]. Also, in this study, changes in the seroprevalence time of populations were presented first. On the other hand, one of the weaknesses of the research was the lack of a sample study from all people and countries of the world to better estimate global seroprevalence. Also, some countries had only one study on the existing cases, and others reported several ones. Indeed, the prevalence of Covid-19 varies in different subgroups and varies according to epidemic changes and prevention policies. Therefore, with a small number of studies, the demographic and temporal generalizability of the findings is problematic. Also, different sampling methods, tests, different times passed from the onset of symptoms in different people, and other antigens make it challenging to interpret the findings uniformly. The probability of underestimating seroprevalence in the world is high. If the prevalence is higher with confirmed cases, a lower death rate can be found in all cases of infection [26]. According to the findings of the studies, the highest prevalence was seen in ethnic and racial minorities such as Blacks and South Asians than Whites. Factors related to this finding include various determinants of health inequality, including discrimination, access to health care, the employment status and its related factors, financial and educational gaps, the housing status and the number of household members, and in general, occupational, social, and environmental variables [37–39].

## Conclusion

The present research performed on 88 studies showed that the seroprevalence of Covid-19 has been between 3 and 15% worldwide, and even considering the low estimate of this rate and the increasing vaccination in the world, a large number of people are still susceptible to Covid-19. Countries need to implement prevention policies with greater sensitivity and follow-up, especially those with low Covid-19 serology prevalence and vaccination coverage.

## Data Availability

Input data for the analyses are available from the corresponding author on request.

## Abbreviations

WHO: World Health Organization
SARS-CoV-2: Severe acute respiratory syndrome coronavirus 2
RT-PCR: Real-time reverse transcription-polymerase chain reaction
PCR: Polymerase chain reaction
Nab: Neutralizing antibodies
LFIA: Lateral flow immunoassays
ELISA: Enzyme-linked immunoassays
FIA: Fluorescence immunoassays
CLIA: Chemiluminescence assays
PsVN: Pseudo-virus neutralization assays
VN: Virus neutralization assays
PRISMA: Preferred Reporting Items for Systematic Reviews and Meta-analyses
JBI: Joanna Briggs Institute
CI: Confidence interval
CINAHL: Cumulative Index to Nursing and Allied Health Literature
EMBASE: Excerpta Medica dataBASE
